# Regional scientific research benefits threatened-species conservation

**DOI:** 10.1093/nsr/nwz090

**Published:** 2018-07-17

**Authors:** Yisi Hu, Zhenhua Luo, Colin A Chapman, Stuart L Pimm, Samuel T Turvey, Michael J Lawes, Carlos A Peres, Tien Ming Lee, Pengfei Fan

**Affiliations:** 1 School of Life Sciences, Sun Yat-Sen University, China; 2 School of Life Sciences, Central China Normal University, China; 3 Department of Anthropology and School of Environment, McGill University, Canada; 4 School of Life Sciences, University of KwaZulu-Natal, South Africa; 5 The College of Life Sciences, Northwest University, China; 6 Nicholas School of the Environment, Duke University, USA; 7 Institute of Zoology, Zoological Society of London, Regent's Park, UK; 8 School of Environmental Sciences, University of East Anglia, UK

Although conventional wisdom considers knowledge of threatened species' ecology and status essential for conservation, few studies demonstrate this in a quantitative way across many species and within the same political entity. Here, we evaluated the impacts of scientific research against conservation interventions (including funding) and species-level correlates, accounting for phylogenetic relatedness, on the conservation of 162 threatened mammal species in China. We did so at three levels: global (all scientific papers published on the species), regional (a subset of the global papers that included at least one author from a local organization) and regional conservation-related (a subset of the regional papers that focused only on ecology and conservation). In addition to protected-area coverage and certain biological traits, regional conservation-related research emerged as an important predictor of species recovery. The same was not the case for global research. We should particularly encourage future regional research effort that has direct relevance to specific conservation issues.

## DOES SCIENTIFIC RESEARCH CONTRIBUTE TO SPECIES CONSERVATION?

Biodiversity loss is accelerating [1]. Moreover, extant vertebrate species have declined in abundance by ∼ 25% since 1970 [2]. In spite of a diverse range of conservation interventions, including the establishment of protected areas and wildlife-protection legislation [3], many threatened species continue to decline [4]. Halting declines is a priority.

Scientific research may play a vital role in conserving threatened species in at least two important ways. First, research provides knowledge about species' biology, ecology and life history, identifies critical limiting resources and determines the relative importance of threats to species. This, in turn, guides appropriate conservation action. Second, scientific research focuses research attention and public awareness, and generates support for conservation from stakeholders and the wider public (e.g. the chimpanzee, *Pan troglodytes*) [5]. However, research may be decoupled from practical conservation intervention, leading to competition for limited resources between scientists and conservation practitioners. For example, some species (e.g. the Yangtze River dolphin, *Lipotes vexillifer*) have been ‘monitored to almost certain extinction' without effective conservation intervention [6]. A critical assessment of the efficacy of scientific research to the conservation of threatened species is required.

## DEPENDENT VARIABLE

Here, we assess the relative importance of scientific research, as indexed by the number of publications to mammal-species conservation in China. We included terrestrial mammals evaluated as Critically Endangered (CR), Endangered (EN) or Vulnerable (VU) in either China's 2004 [7] or 2015 Species Red List [8]. These Red Lists were conducted by Chinese scientists following IUCN Guidelines (version 4.0) and using IUCN Red List Categories and Criteria (version 8.1). More information of these assessments can be found in the Supplementary file. We believe that these extensive and robust national assessments represented a real status change of the Chinese mammal species, and are unlikely to be caused by more information being available. We calculated the change in status score for each species by converting species' status to a numerical index, i.e. 0 (Least Concern), 1 (Near Threatened), 2 (Vulnerable), 3 (Endangered) and 4 (Critically Endangered), following previous studies [9]. We subtract the species' 2015 status score from their 2004 score, such that a positive score indicates a species has become less threatened. The full species list and status-change score are provided in Supplementary Table 1.

**Figure 1.**
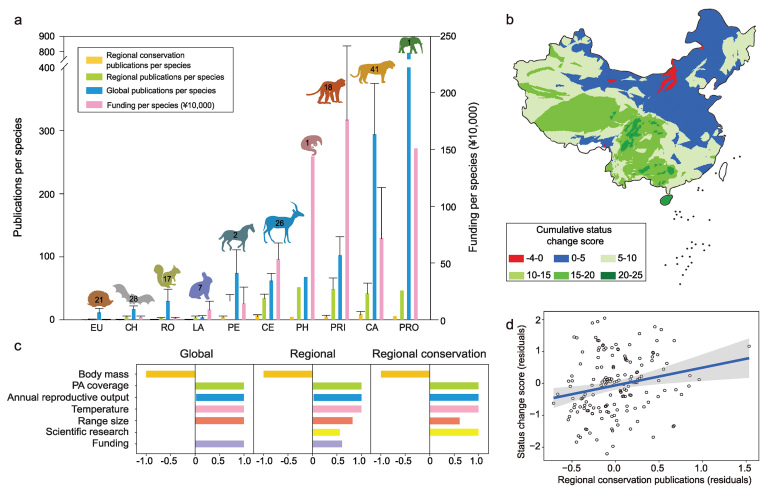
(a) Distribution of publications and funding (mean and SD) for species in different mammalian orders in China. EU, Eulipotyphla; CH, Chiroptera; RO, Rodentia; LA, Lagomorpha; PE, Perissodactyla; CE, Cetartiodactyla; PH, Pholidota; PRI, Primates; CA, Carnivora; PRO, Proboscidea. Each column with a different colour represents the number of publications or funding allocated to that order. Numbers indicate the number of species in each order included in this study. (b) Geographical distribution of status-change scores in China. A value of +1 means an improvement of one class, e.g. from Endangered to Vulnerable; scores sum changes across all species that occur in a region. (c) Final ‘best-fitted' models using global, regional or regional conservation-related publications to represent scientific research effort. Lengths of bars indicate the relative importance of variables in different cases, and directions indicate either positive or negative impact on the response variable. (d) Partial regression plots showing the relationship between species recovery and regional conservation-relevant scientific research, with 95% confidence intervals.

## PREDICTOR FACTORS

Based on previous research, we selected 10 predictor factors including three intrinsic factors (body mass, generation length and annual reproductive output), four ecological factors (species range size, temperature, precipitation and human-footprint index of species distribution area) and three conservation interventions (protected-area coverage, number of publications and research funding on each species) (Supplementary Table 2). We extracted the life-history information of species from the several high-quality databases (see Supplementary Methods) and digitized their distribution maps from China's mammal-diversity and geographical-distribution dataset [10]. We converted the polygon map of each species into a raster map on a 1-km^2^ equal-area grid scale. For each species, we first computed its range size and then obtained the mean value of annual temperature, precipitation [11] and Human Footprint Index (HFP) [12] across its distribution area.

To derive an index of the amount of scientific research allocated to each species, we determined the number of publications for each species from literature databases for the entire duration of their records. It is possible that we did not include some relevant unpublished publications in our indices, as these reports may not be indexed in the databases. For each species, the total number of publications represented the global research effort, while the number of publications including at least one author from a Chinese institute in the list of author affiliations represented regional research effort. We assumed that having a regional author would make species-conservation action more likely. A more direct way would be to track the conservation influence of each paper but such information is not readily available. We filtered the regional publications for ‘Biodiversity & Conservation' and ‘Environmental Science & Ecology' to determine the number of regional conservation-related publications (see Supplementary Methods for details). We determined the amount of research funding assigned to each species from the National Natural Science Foundation of China (http://www.nsfc.gov.cn/) (see Supplementary Methods for details).

Protected-area coverage for each species was obtained from the World Database of Protected Areas, supplemented with data from Wu *et al.* [13] and the Chinese Ministry of Environmental Protection. We expressed the conservation effort allocated to each species at the national scale by calculating the proportion of species' ranges included in national protected areas.

## MODELLING

In the modelling process, we constructed the phylogenetic tree of all species of interest according to Tree of Life [14] (Supplementary Fig. 1) and used phylogenetic generalized linear models for our analyses (Supplementary Table 3). Model selection followed an information-theoretic approach using the corrected Akaike Information Criterion AICc [15]. We calculated the relative importance (*w*+) of the variables in the candidate model set using Akaike weights (*w*_i_) [16]. To explore the relationship between scientific research and species recovery, we performed partial regression with the species status-change score against number of scientific publications, while controlling for the influence of all other variables in the final models (see Supplementary Methods for details).

In China, the funding and number of publications varied considerably across the 162 species (Fig. 1a). The endangerment status of 76 species improved, 59 remained the same and 27 became worse in 2015 compared to 2004. Improvements in the species-conservation status were most obvious in south-west China, where regional mammal diversity is highest [10]. In contrast, the species-conservation status in north-east China worsened or did not change (Fig. 1b).

## PROTECTED AREA AND OTHER FACTORS

The best-fit phylogenetic generalized linear models (PGLM) models testing the effect of regional publications included six or seven predictor variables (Supplementary Table 3 and Fig. 1c). Excluding the giant panda, which was a major outlier, did not affect our main results (not shown). Consistently with previous studies, our study found that conservation interventions, including the establishment of protected areas [3] and funding allocation [17], were associated with reversing population declines of threatened species, though the effects may differ for different publication indices (Supplementary Table 4 and Fig. 1c). Protected areas maintain relatively intact habitats, reduce human disturbance and suppress hunting [18]. In addition, species occupying larger geographic ranges and warmer environments had a greater likelihood of recovery. Large-bodied mammals with slow life histories became more threatened over time (Supplementary Table 4 and Fig. 1c).

## REGIONAL RESEARCH BENEFITS SPECIES CONSERVATION

Our analyses suggest that regional scientific research including local authors, especially regional conservation-related research, was an important predictor of improved species status (Supplementary Table 4 and Fig. 1c and d). Although this relationship is correlational, we believe that increased research effort may have caused improvements to the status of some species. It is unlikely that species recovery caused more research, since conservation scientists usually target species that are experiencing population decline and not those in recovery. Species with deteriorating status should cause more, not less, research, which would result in a negative correlation, and that is opposite to what we found.

Conservation research of threatened mammals in China has seen considerable growth in the last two decades [19], although the number of publications is still small (average of 4.2 regional conservation-related papers per species). When few data are available for threatened species, even a small number of conservation-related research projects can contribute to conservation in at least three ways. First, research can provide crucial knowledge of species biology, ecology, behaviour and threats, thus informing and promoting species conservation. Second, research can attract public attention. When we described and published a new species of gibbon (the skywalker hoolock gibbon, [20]), it attracted >400 media reports in Chinese and English. Google hits increased rapidly after the paper was published (Supplementary Fig. 2). Third, regional research can affect policy making. Chinese scientists were involved in the conservation planning of the Giant Panda National Park since the pre-planning stage. Scientific research of tigers and leopards in north-east China directly led to the Central Government of China establishing the 14 600-km^2^ Northeast Tiger Leopard National Park in 2017 (tiger.gov.cn). Our results reject concerns that scientific research is irrelevant to, or potentially disconnected from, practical conservation. The exact mechanisms of how research contributes to conservation will require further investigation.

## GLOBAL RESEARCH DID NOT PREDICT SPECIES RECOVERY

The number of global publications and species status change in China were not correlated (Fig. 1c, global panel). While global studies may provide useful knowledge to support regional conservation activities for threatened species, our analyses suggest that regional research is more predictive and more useful in promoting population recovery. This is because species in different regions often face different and contextualized ecological and anthropogenic threats. In addition, regional scientists are more likely to become involved in local conservation activities. Our findings point to the important role local and regional conservation organizations have in threatened-species research and conservation actions.

In conclusion, our study provides correlational evidence that scientific research, especially directed regional conservation-related research, plays an important role in successful species conservation, although the exact mechanisms remain to be examined. In the future, promoting regional research that has direct relevance to specific conservation issues and species should be encouraged and funded.

## Supplementary Material

nwz090_Supplemental_Files
