# Supplementary material for: Regional scientific research benefits threatened-species conservation
Source: Natl Sci Rev. 2018 Jul 17;6(6):1076–9. doi: 10.1093/nsr/nwz090 (PMC8291549; doi:10.1093/nsr/nwz090)
Supplement: nwz090_Supplemental_Files [file nwz090_supplemental_files.zip › supplementary_materials_and_methods-0623_nwz090.docx]

**Supplementary Materials and Methods**

**Background of the China’s Species Red List**

Professors Song Wang and Yan Xie published China’s Species Red List in 2004 [1] according to the IUCN Red List Categories and Criteria (Version 3.1), and Guidelines for Application of IUCN Red List Criteria at Regional and National Levels (Version 4.0). The authors explained the evaluation process and how to use this Red List in Volume I. The endangerment status, population dynamic, criteria used, and justifications for all mammal species are available in Part 2 of Volume II. More than 100 experts were involved in this assessment (from the forward of this series of book). We believe in the integrity of the data in these reports.

From 2013 to 2015, the Ministry of Environmental Protection collaborated with Chinese Academy of Sciences compiled the Red List of China’s vertebrates according to IUCN Red List Categories and Criteria (Version 3.1), Guidelines for Using the IUCN Red List Categories and Criteria (Version 8.1), and Guidelines for Application of IUCN Red List Criteria at Regional and National Levels (Version 4.0). This second assessment included the following steps: information collection, database establishment, formation of an advisory committee, core experts group, and working group, and establishment of a pool of corresponding experts. The Red List was evaluated and officially released in the form of the 32nd Announcement of the Ministry of Environmental Protection of the P.R. China and the Chinese Academy of Sciences on May 23, 2015 (available at http://english.mep.gov.cn). The Red List provided criteria and justifications for each species. Prof. Zhigang Jiang and his colleagues published a paper about the assessment and evaluation processed [2]. This paper also provided a list of 61 Chinese mammalogists that were involved in the assessment. From the list, we can see nearly all senior Chinese mammalogists were involved.

These two nation-wide assessments used the exact same IUCN categories and criteria, and are the most comprehensive assessment of Chinese mammal species ever carried out. We believe that these assessments captured real status change of Chinese mammal species, and are not caused by more information being available. In addition, they are the only Red List assessments we can obtain and use for this kind of study.

**Species list**

We examined threatened terrestrial mammals because: extensive data are available on the biology, ecology, and phylogeny of mammals, and unlike birds and marine mammals, terrestrial mammals typically do not migrate over long distances, and are therefore subject to local or regional conservation efforts.

Following the IUCN Red List Categories and Criteria, vertebrate species in China were evaluated and classified into nine levels of threat status, among which Critically Endangered (CR), Endangered (EN) and Vulnerable (VU) are considered as “Threatened Categories”. We focused on terrestrial mammals included within “Threatened Categories” at least once in the two CSRL assessments. We excluded those species for which the status was Data Deficient (DD) in either evaluation. We also excluded species for which there was a taxonomic change that could have affected the interpretation of threat status from the dataset (for example, if a single species was split into several new species). We excluded Taiwan and Southeast Tibet and their endemics because these regions have different conservation systems. The full species list is provided in Table S1 (a separate excel file).

**Literature search**

Global research refers to all publications on our target species that were included in the database, regardless of study area or specific subject. We considered that the number of publications involved Chinese organizations (at least one Chinese institute in the list of author affiliations) represented an index of regional research effort, which is a subset of global research. We assumed that studies related to biodiversity conservation, ecology, and/or environmental science could have more positive effects on species conservation than other types of research, so we then refined the research area to “Biodiversity & Conservation” or “Environmental Sciences & Ecology” within regional research to represent an additional category of regional conservation-related research, which is a subset of regional research.

For Chinese literature, we included Chinese publications from three mutually exclusive literature databases. We searched the Chinese Science Citation Database (<http://www.webofknowledge.com>) for Chinese journal articles. We used the Chinese formal names or scientific names, with quotation marks, as the searching terms and searched by “topic”. We refined the research area to “Biodiversity & Conservation” or “Environmental Sciences & Ecology” to represent conservation-related researches. For Chinese theses and dissertations, we searched the CAS Thesis & Dissertation Database (http://dpaper.las.ac.cn) and the CNKI China Academic Journals Full-text database (http://[cnki.net](file:///C:\Users\tienminglee\Documents\WeChat%20Files\AppData\Local\AppData\工作\2016.10.12工作备份\研究物种\Review%20and%20meta%20analysis\中国野生濒危哺乳动物的保护\MS\cnki.net)), which are mutually exclusively. We searched the CAS Thesis & Dissertation Database with species’ Chinses formal names or scientific names as keywords, and determined the research as conservation-related if the word “conservation” (in Chinese) is mentioned in the abstract, but not including those only mention “conservation area” as their study sites or other unrelated topic. And we searched CNKI China Academic Journals Full-text database using species’ Chinese formal names or scientific names by “topic”. We refined the publications according to CNKI categories, including “Wildlife Conservation & Cultivation”, “Forest Conservation”, “Environmental Sciences”, and “Environmental Sciences & Engineering” as conservation-related. We regarded all these publications in Chinese as regional research. We excluded studies that focused on medical experiments, pharmacology, and animal husbandry by reading the title, abstract and/or keywords in the regional dataset. Here, we excluded *Macaca mulatta* from the analysis of China due to the extensive use of this species as a biomedical model animal.

For English literature, we searched the Web of Science Core Collection (<http://www.webofknowledge.com>) for journal papers and the ProQuest Dissertations & Theses Global (https://search.proquest.com) for dissertations and theses. When searching the Web of Science, we used the species’ scientific name as searching term and searched by “topic”, to record the global publications. Then we considered the publications by at least one Chinese organizations in author affiliations as regional research. Similarly, we refined the research area to “Biodiversity & Conservation” or “Environmental Sciences & Ecology” from regional research to represent regional conservation-related research. And we determined the conservation-related areas as follows: environment science, wildlife conservation, wildlife management, environmental management, conservation, environmental studies, according to Proquest categories.

**Species distribution map and ecological factors**

Distribution maps of terrestrial mammals were digitized from China's mammal diversity and geographic distribution dataset [3]. We converted the polygon map of each species into a raster map at 1 km^2^ equal-area grid scale. For each species, we first computed its range size within the country and then obtained mean annual temperature and precipitation [4] and Human Footprint Index (HFP) [5]. We rescaled all variables into a 1 km^2^ equal-area grid, and extracted their means across the distribution of each species using ArcGIS 10.0.

**Protected area**

When calculating the protected area coverage for each species, we included 809 national protected areas in the analysis. Although there are other different levels of protected areas in China (provincial, municipal, and prefectural protected areas), we considered the national management efforts are most reliable [6].**Funding**

To evaluate research funding in China, we accessed one funding resource from the National Natural Science Foundation of China (http://www.nsfc.gov.cn/). This was established in 1986 and is the main source of research funding for Chinese scientists and the only one to which we had access. As with the analysis of publications, we counted the total number of projects in ecology, zoology, and genetics and excluded those on biomedicine and pharmacology, animal husbandry, and non-ecological related subjects. For projects targeting more than one species, we divided the total number and amount of funds of the project equally among all species to which it referred.

**Traits and Phylogeny**

We used a random forest approach to impute missing data of all biological traits (package missForest [7]) in R [8] following [9] and evaluated the accuracy of imputation using the out-of-bag error rate. We extracted all species of interest from the Tree of Life [10] and tested the phylogenetic signal in our data structure against the assumption of random evolution (Brownian motion) (Fig. S1). Given the presence of a phylogenetic signal, we used phylogenetic generalized linear models (PGLM, “caper” package [11]). Performance of our models were shown in Table S3.

**Modeling**

Model selection followed an information-theoretic approach, using the corrected Akaike Information Criterion AICc [12]. Body mass, generation length, range size distribution, number of scientific publications, and funding were all log-transformed, while protected area coverage was square root-transformed. We calculated the variance inflation factor (VIF) to check for multicollinearity of the explanatory variables. Because VIF < 10 for all variables in the CSRL dataset, we did not exclude any variables from subsequent analysis. We compared all combinations of predictor variables (i.e. competing models) using the “dredge” function in the MuMIn package [13], and selected those models with ΔAICc<2 as the ‘best-fit’ models. We calculated the relative importance (*w*+) of the variables in the candidate model set using Akaike weights (*w*_i_) [14].

**Figure S1** The phylogenetic tree of the 162 species covered by the analysis.
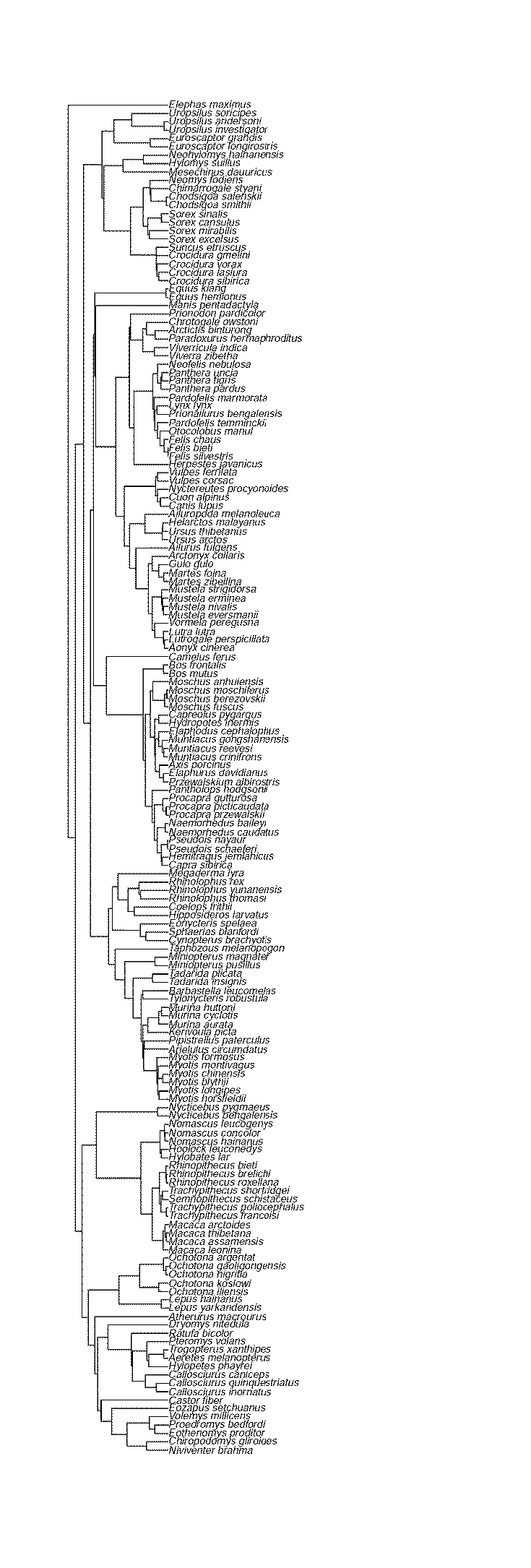


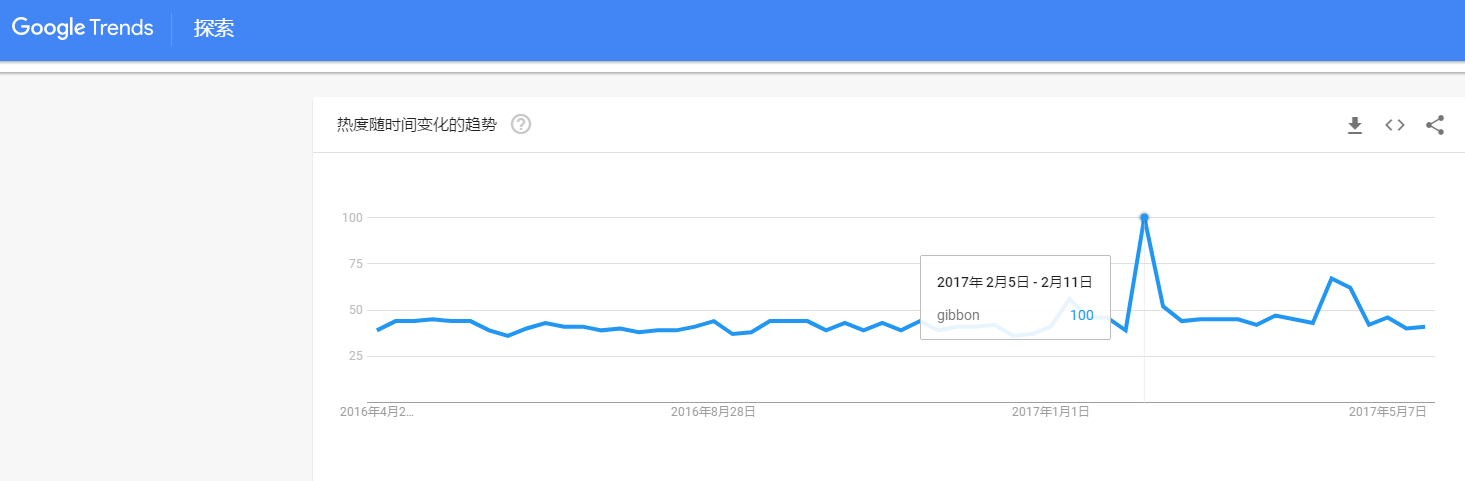


Figure S2. Google trend of term “gibbon” from April 2016 to May 2017. Please note the peak of searching appeared in the week when a paper describing a new species of gibbon was published in American Journal of Primatology (Fan et al., 2017).

.

Table S2. Summary of the explanatory variables and hypothesized relationship considered in the models. Examples of previous work examining the variables are provided in the Reference column.

| **Variable** | **Justification and Hypothesized Relationship** | **Data sources** | **Reference** |
| --- | --- | --- | --- |
| Body mass | Larger, k-selected species usually have higher risk of extinction; negative relationship with population change | An Age [15];  Generation length for mammals [16];  Handbook of Mammals of the World series [17–20] | [21–25] |
| Generation length | Species with longer generation length respond more slowly to conservation effort; negative relationship with population change | Generation length for mammals [16] | [21,25] |
| Annual reproductive output | Species reproducing at higher rate recovery faster; positive relationship with population change | PanTHERIA [26];  AnAge [15];  Handbook of Mammals of the World series [17–20] | [22,25,27] |
| Range size | Widespread species are more likely to recover; positive relationship with population change | *China's mammal diversity and geographic distribution*[3]; | [21,27,22–24] |
| Temperature | Species in areas with higher productivity metabolize faster; positive relationship with population change | Hijmans, et al.[4] | [22] |
| Precipitation | Species in areas with higher productivity metabolize faster; positive relationship with population change | Hijmans, et al. [4] | [22] |
| PA coverage | Larger percentage of species range size covered by PA represents more protection species receive; positive relationship with population change | World Database of Protected Areas [28];  Wu, et al. [29]  The List of Protected Areas in China [30] | [31] |
| Human Footprint Index (HFP) | Species suffering from severer human disturbance may decrease more quickly in population; negative relationship with population change | Global human influence index (HII) Dataset [5,32] | [33,27,22] |
| Number of publications | Scientific research may facilitate species conservation; positive relationship with population change | Chinese Science Citation Database (<http://www.webofknowledge.com>);  CAS Thesis & Dissertation Database (http://dpaper.las.ac.cn);  CNKI China Masters' Theses Full-text Database and China Doctoral Dissertations Full-text Database (http://[cnki.net](file:///C:\Users\tienminglee\Documents\WeChat%20Files\AppData\Local\AppData\工作\2016.10.12工作备份\研究物种\Review%20and%20meta%20analysis\中国野生濒危哺乳动物的保护\MS\cnki.net));  Web of Science (<http://www.webofknowledge.com>);  ProQuest Dissertations & Theses Global (https://search.proquest.com) | Tested by this research |
| Funding | Funding may provide practical help to species conservation; positive relationship with population change | National Natural Science Foundation of China (http://www.nsfc.gov.cn/); | [34] |

**Table S3** Model performance of ‘best-fitted’ models. (a) Models fitted using global publications to represent all scientific research. (b) Models fitted using regional publications to represent regional scientific research. (c) Models fitted using regional conservation-related publications to represent regional conservation research. (Significance: *** *p*-value<0.001, ** *p*-value<0. 01, * *p*-value<0.05)

| Intercept | Body mass | Funding | PA coverage | Range size | Annual reproductive output | Temperature | ΔAICc | adj. R^2^ | |
| --- | --- | --- | --- | --- | --- | --- | --- | --- | --- |
| -1.306 | -0.255** | 0.232* | 1.619* | 0.245* | 0.096* | 0.030* | 0 | 0.111 |  |

(b)

| Intercept | Body mass | Funding | PA coverage | Range size | Regional publications | Annual reproductive output | Temperature | ΔAICc | adj. R^2^ |
| --- | --- | --- | --- | --- | --- | --- | --- | --- | --- |
| -1.306 | -0.255** | 0.232* | 1.619* | 0.245* | / | 0.096* | 0.030* | 0 | 0.111 |
| -0.983 | -0.265** | / | 1.759* | 0.179 | 0.275* | 0.093* | 0.027* | 1.484 | 0.114 |
| -0.013 | -0.252** | / | 1.573* | / | 0.349* | 0.100* | 0.020 | 1.785 | 0.100 |
| -1.219 | -0.265** | 0.186 | 1.683* | 0.221 | 0.102 | 0.098* | 0.030* | 1.958 | 0.108 |

(c)

| Intercept | Body mass | Funding | PA coverage | Range size | Regional conservation-related publications | Annual reproductive output | Temperature | ΔAICc | adj. R^2^ |
| --- | --- | --- | --- | --- | --- | --- | --- | --- | --- |
| -0.934 | -0.289** | / | 1.848** | 0.173 | 0.593** | 0.098* | 0.031* | 0.000 | 0.143 |
| 0.046 | -0.271** | / | 1.631* | / | 0.656*** | 0.103* | 0.024 | 0.375 | 0.131 |
| -1.003 | -0.292** | 0.062 | 1.833** | 0.182 | 0.525* | 0.101* | 0.032* | 1.979 | 0.136 |

**Table S4** Final models for China built on ‘best-fitted’ models according to AICc. All models were constructed using phylogenetic generalized linear regression, fitted by maximum likelihood. The relative variable importance was calculated using Akaike weights. (a) Models fitted using global publications to represent all scientific research. (b) Models fitted using regional publications to represent regional scientific research. (c) Models fitted using regional conservation-related publications to represent regional conservation research.

(a)

|  | Estimate | Std. Error | z value | *p* value | Relative Variable Importance |
| --- | --- | --- | --- | --- | --- |
| (Intercept) | -1.306 | 0.811 | -1.611 | 0.109 |  |
| Body mass | -0.255 | 0.090 | -2.819 | 0.006 | 1.00 |
| PA coverage | 1.619 | 0.666 | 2.430 | 0.016 | 1.00 |
| Annual reproductive output | 0.096 | 0.041 | 2.333 | 0.021 | 1.00 |
| Temperature | 0.030 | 0.013 | 2.279 | 0.024 | 1.00 |
| Range size | 0.245 | 0.110 | 2.233 | 0.027 | 1.00 |
| Funding | 0.232 | 0.106 | 2.186 | 0.030 | 1.00 |

(b)

|  | Estimate | Std. Error | z value | *p* value | Relative Variable Importance |
| --- | --- | --- | --- | --- | --- |
| (Intercept) | -0.989 | 0.902 | 1.097 | 0.273 |  |
| Body mass | -0.258 | 0.090 | 2.853 | 0.004 | 1.00 |
| PA coverage | 1.651 | 0.677 | 2.439 | 0.015 | 1.00 |
| Annual reproductive output | 0.097 | 0.041 | 2.344 | 0.019 | 1.00 |
| Temperature | 0.028 | 0.014 | 2.007 | 0.045 | 1.00 |
| Range size | 0.223 | 0.117 | 1.915 | 0.056 | 0.82 |
| Funding | 0.219 | 0.119 | 1.849 | 0.064 | 0.61 |
| Regional publications | 0.247 | 0.198 | 1.253 | 0.210 | 0.56 |

(c)

|  | Estimate | Std. Error | z value | *p* value | Relative Variable Importance |
| --- | --- | --- | --- | --- | --- |
| (Intercept) | -0.577 | 0.849 | 0.679 | 0.497 |  |
| Body mass | -0.283 | 0.088 | 3.219 | 0.001 | 1.00 |
| Regional conservation-related publications | 0.605 | 0.209 | 2.899 | 0.004 | 1.00 |
| PA coverage | 1.764 | 0.670 | 2.632 | 0.009 | 1.00 |
| Annual reproductive output | 0.100 | 0.040 | 2.495 | 0.013 | 1.00 |
| Temperature | 0.029 | 0.013 | 2.155 | 0.031 | 1.00 |
| Range size | 0.176 | 0.111 | 1.588 | 0.112 | 0.62 |

**References:**

1. Wang S, Xie Y. *China Species Red List, Vol. 1: Red List*. Beijing, China: Higher Education Press, 2004.

2. Jiang Z, Li LL, Luo ZH *et al.* Evaluating the status of China's mammals and analyzing their causes of endangerment through the red list assessment. *Biodiversity Science* 2016; **24**:552–67.

3. Jiang Z, Ma Y, Wu Y *et al.* eds. *China’s Mammal Diversity and Geographic Distribution*. Beijing, China: Science Press, 2015.

4. Hijmans RJ, Cameron SE, Parra JL *et al.* Very high resolution interpolated climate surfaces for global land areas. *Int J Climatol* 2005;**25**:1965–78.

5. Venter O, Sanderson EW, Magrach A *et al.* Global terrestrial Human Footprint maps for 1993 and 2009. *Scientific Data* 2016;**3**:160067.

6. Luo Z, Wei S, Zhang W *et al.* Amphibian biodiversity congruence and conservation priorities in China: Integrating species richness, endemism, and threat patterns. *Biological Conservation* 2015;**191**:650–8.

7. Stekhoven DJ, Buhlmann P. MissForest--non-parametric missing value imputation for mixed-type data. *Bioinformatics* 2012;**28**:112–8.

8. R Core Team. *R: A Language and Environment for Statistical Computing.* Vienna, Austria: R Foundation for Statistical Computing, 2018.

9. Penone C, Davidson AD, Shoemaker KT *et al.* Imputation of missing data in life-history trait datasets: which approach performs the best? *Methods in Ecology and Evolution* 2014;**5**:961–70.

10. Hedges SB, Marin J, Suleski M *et al.* Tree of Life Reveals Clock-Like Speciation and Diversification. *Molecular Biology and Evolution* 2015;**32**:835–45.

11. Orme D, Freckleton R, Fritz S *et al.* *Caper: Comparative Analyses of Phylogenetics and Evolution in R.*, 2018.

12. Burnham KP, Anderson DR. *Model Selection and Multimodel Inference: A Practical Information-Theoretic Approach*. 2nd ed. New York: Springer, 2002.

13. Bartoń K. *MuMIn: Multi-Model Inference.*, 2018.

14. Burnham KP, Anderson DR, Huyvaert KP. AIC model selection and multimodel inference in behavioral ecology: some background, observations, and comparisons. *Behavioral Ecology & Sociobiology* 2011;**65**:23–35.

15. Tacutu R, Craig T, Budovsky A *et al.* Human Ageing Genomic Resources: Integrated databases and tools for the biology and genetics of ageing. *Nucleic Acids Research* 2013;**41**:D1027–33.

16. Pacifici M, Santini L, Di Marco M *et al.* Generation length for mammals. *Nature Conservation* 2013;**5**:89–94.

17. Wilson DE, Mittermeier RA eds. *Handbook of the Mammals of the World-Volume 1 Carnivores*. Barcelona, Spain: Lynx Edicions, 2009.

18. Wilson DE, Mittermeier RA eds. *Handbook of the Mammals of the World-Volume 2 Hoofed Mammal*. Barcelona, Spain: Lynx Edicions, 2011.

19. Mittermeier RA, Rylands AB, Wilson DE eds. *Handbook of the Mammals of the World-Volume 3, Primates*. Barcelona, Spain: Lynx Edicions, 2013.

20. Wilson DE, Lacher TEJr, Mittermeier RA. *Handbook of the Mammals of the World-Volume 6 Rodent Ⅰ.* Barcelona, Spain: Lynx Edicions, 2016.

21. Purvis A, Gittleman JL, Cowlishaw G *et al.* Predicting extinction risk in declining species. *Proceedings of the Royal Society B: Biological Sciences* 2000;**267**:1947–52.

22. Cardillo M, Mace GM, Gittleman JL *et al.* The predictability of extinction: biological and external correlates of decline in mammals. *Proceedings of the Royal Society B: Biological Sciences* 2008;**275**:1441–8.

23. Fritz SA, Bininda-Emonds ORP, Purvis A. Geographical variation in predictors of mammalian extinction risk: big is bad, but only in the tropics. *Ecology Letters* 2009;**12**:538–49.

24. Davidson AD, Hamilton MJ, Boyer AG *et al.* Multiple ecological pathways to extinction in mammals. *Proceedings of the National Academy of Sciences* 2009;**106**:10702–5.

25. González-Suárez M, Revilla E. Variability in life-history and ecological traits is a buffer against extinction in mammals. Arita H (ed.). *Ecology Letters* 2013;**16**:242–51.

26. Jones KE, Bielby J, Cardillo M *et al.* PanTHERIA: a species-level database of life history, ecology, and geography of extant and recently extinct mammals: *Ecological Archives* E090-184. Michener WK (ed.). *Ecology* 2009;**90**:2648–2648.

27. Cardillo M. Multiple Causes of High Extinction Risk in Large Mammal Species. *Science* 2005;**309**:1239–41.

28. UNEP-WCMC. World database on protected areas (WDPA). 2016.

29. Wu R, Zhang S, Yu DW *et al.* Effectiveness of China’s nature reserves in representing ecological diversity. *Frontiers in Ecology and the Environment* 2011;**9**:383–9.

30. China Ministry of Environmental Protection. The list of protected areas in China. 2016.

31. Gray CL, Hill SLL, Newbold T *et al.* Local biodiversity is higher inside than outside terrestrial protected areas worldwide. *Nature Communications* 2016;**7**:12306.

32. CIESIN, Wildlife Conseravtion Society. Last of the wild project, version 2, 2005 (LWP-2): Global human influence index (HII) dataset (geographic). 2005.

33. Cardillo M, Purvis A, Sechrest W *et al.* Human Population Density and Extinction Risk in the World’s Carnivores. Craig Moritz (ed.). *PLoS Biology* 2004;**2**:e197.

34. Waldron A, Mooers AO, Miller DC *et al.* Targeting global conservation funding to limit immediate biodiversity declines. *Proceedings of the National Academy of Sciences* 2013;**110**:12144–8.
